# Entropy Churn Metrics for Fault Prediction in Software Systems

**DOI:** 10.3390/e20120963

**Published:** 2018-12-13

**Authors:** Arvinder Kaur, Deepti Chopra

**Affiliations:** University School of Information and Communication Technology (U.S.I.C.T), Guru Gobind Singh Indraprastha University, New Delhi 110087, India

**Keywords:** fault prediction, entropy, mining software repositories, software metrics

## Abstract

Fault prediction is an important research area that aids software development and the maintenance process. It is a field that has been continuously improving its approaches in order to reduce the fault resolution time and effort. With an aim to contribute towards building new approaches for fault prediction, this paper proposes Entropy Churn Metrics (ECM) based on History Complexity Metrics (HCM) and Churn of Source Code Metrics (CHU). The study also compares performance of ECM with that of HCM. The performance of both these metrics is compared for 14 subsystems of 5different software projects: Android, Eclipse, Apache Http Server, Eclipse C/C++ Development Tooling (CDT), and Mozilla Firefox. The study also analyses the software subsystems on three parameters: (i) distribution of faults, (ii) subsystem size, and (iii) programming language, to determine which characteristics of software systems make HCM or ECM more preferred over others.

## 1. Introduction

With growing research in the field of software engineering, many fault prediction approaches have been developed. Predicting faults in software helps software developers and maintainers to focus on more fault-prone entities. More time and effort is expended on fault-prone software components during software development and the maintenance process. Software metrics are used to measure the degree to which a software system possesses a certain characteristic [1]. Fault prediction is also usually based on certain characteristics of the software development process (i.e., software metrics are used to predict faults in a software system). For instance, Chidamber and Kemerer (CK) metrics are a set of popular software metrics used for fault prediction [2]. Also, Khoshgoftaar et al. devised a metric suite for prediction offault-prone modules based on the amount of past modifications done in the code files [3]. Bernstein et al. employed temporal features such as number of changes and number of reported issues for predicting number of faults in future releases of the software [4]. 

There are many software metrics proposed for predicting faults in software systems and D’Ambros et al. conducted a broad comparison of popular fault prediction approaches, namely, Change Metrics, Previous Defects, Source Code Metrics, Entropy of Changes, Churn of Source Code Metrics, and Entropy of Source Code Metrics [5]. D’Ambros et al. extended their study and provided an open source benchmark dataset for fault prediction, for evaluation of a variety of fault prediction approaches [6]. The benchmark dataset consisted of metrics for five open source software systems. Source code metrics and churn of source code metrics are both popular metrics for fault prediction. Churn of source code metrics were shown to perform better than source code metrics for fault prediction [6]. Entropy of software system is another metric which is used for fault prediction, but the concept of churn of entropy has not yet been proposed and evaluated. With this motivation, this paper proposes Entropy Churn Metrics (ECM) for prediction of faults in software systems. Its performance is compared with History Complexity Metrics (HCM), which model entropy of changes in software subsystems [7]. Both these metrics are based on the concept of entropy of software change. HCM is based on absolute value of entropy, whereas ECM models the change in entropy. In this study, ECM is evaluated to determine whether churn-of-entropy-based metrics can be used for efficient prediction of faults in a software system. The study also aims to determine when it is better to use churn of entropy (ECM) over entropy (HCM) for predicting faults in a software subsystem and vice versa. The fault prediction performance of both these metrics is evaluated on 14 software subsystems from 5software projects, namely Android, Eclipse, Apache Http Server, Eclipse C/C++ Development Tooling (CDT), and Mozilla Firefox. The software subsystems chosen for the study are of different sizes, are written in different programming languages, and have varying distribution of faults. This would help in analyzing and determining the tendency of certain types of subsystems to favor a particular metric over another. 

The rest of the paper is organized as: Section 2 discusses related work in the area of fault prediction and entropy; Section 3 proposes ECM and describes other related metrics. Research materials and methods are described in Section 4 and results are presented and analyzed in Section 5. Results are discussed in Section 6, while Section 7 discusses threats to the validity of the study. Finally, in Section 8, the study is concluded.

## 2. Related Work

Entropy is used to measure the degree of randomness or uncertainty in the system. Apart from thermodynamics and information theory, entropy measures are being used in a variety of other areas. Gou et al. have used hesitant fuzzy linguistic entropy and cross-entropy measures for multiple criteria decision making [8]. The hesitant fuzzy linguistic entropy and cross-entropy measures are used to determine the weights for the multiple criteria, so that a ranking of alternatives is obtained. Pramanik et al. have also used cross-entropy measures of bipolar and interval bipolar neutrosophic sets to develop two approaches for multiattribute decision making [9]. Entropy measures also have applications for water monitoring. Keum et al. [10] review and summarize the applications of entropy for water monitoring network design, such as usage of entropy weight method to measure the water quality [11]. Wu et al. [12], employ a joint entropy-based learning model for optimizing image retrieval. Thus entropy has applications in many fields, including fields like the stock market [13]. In fact, Baldwin has used maximum entropy modelling to determine distribution and habitat selection for various wildlife species [14]. In this study, an entropy-based metric (i.e., ECM) is proposed to model churn of entropy of software code changes. The proposed metric (i.e., ECM) is used for predicting faults in software systems and its performance is compared with HCM. 

Fault prediction is an active research area in the field of software engineering. Many techniques and metrics have been developed to improve fault prediction performance. D’Ambros et al. have compared popular fault prediction approaches for software systems [6], namely, process metrics [15], previous faults [16], source code metrics [17], entropy of changes [7], churn of source code metrics [18], and entropy of source code metrics [6]. Nagappan and Ball proposed a technique for prediction of defect density of the system using relative code churn measures and compared its performance with absolute churn metrics [19]. Hassan proposed absolute entropy metrics or HCM that modelled the complexity of code changes and validated its use for fault prediction [7]. In this study, it is proposed to extend entropy metrics and predict faults on the basis of change in entropy (ECM) rather than absolute value of entropy (HCM). The concept of churn of entropy had not been established earlier, thereby making its evaluation essential to fault prediction research.

Fault prediction is based not only on the current characteristics of the software, but on the entire software evolution history. Historical data stored in software repositories has been time and again used for fault prediction. For instance, Raja et al. presented a time series analysis of software faults from eight different open source software (OSS) projects [20]. It was found that the time series model accurately predicted the software evolution faults for all of the eight OSS projects. Wu et al. also conducted a study that used time series analysis for prediction of fault numbers [21]. The study compared three different approaches for time series analysis on bug data from Debian software. Faults are closely related to changes made in the software systems and studying the changes that take place during software evolution is also important. Yazdi et al. studied the evolution of changes in the software systems using reverse-engineered class diagrams from nine open source Java projects [22]. It was observed that only discrete Pareto, Waring, Yule, and Beta-Negative Binomial distributions were able to satisfactorily describe the observed evolution of changes. Further, Yazdi et al. proposed a framework that captured the changes between model revisions [23]. Forecasting and simulation performance of different time series models were also tested. In this study also, the software changes are analyzed to compute ECM and HCM that are used for predicting faults.

Entropy-based measures have been used for various purposes in software engineering research. For instance, Trienekens et al. used the concept of internal and external entropy to model the level of disorder in the system and its environment, and then based process improvement on these suggested directions [24]. Allen et al. also proposed measures for size, complexity, and coupling of software using entropy and information theory concepts [25]. Ma combined fractal and entropy measures to determine self-similarity and structural ordering in software [26]. Kaur et al. evaluated entropy-based fault prediction using neural-network-based regression [27] and locally weighted regression [28]. Kaur et al. also studied and compared machine learning techniques for entropy-based fault prediction without reference to churn of entropy [29]. In this paper, a new metric Entropy Churn Metric (ECM) based on History Complexity Metric (HCM) is proposed. It takes into account the complexity of code changes for fault prediction and calculates its churn. ECM is calculated using HCM similar to Source Code Churn Metrics [18], which calculated the churn of source code metrics.

Researchers have been conducting studies to review and compare existing fault prediction approaches. Comparison of approaches leads to better understanding of performance of various approaches on different types of software projects. Radjenovic et al. conducted a review on fault prediction metrics which identified and analyzed the applicability of various fault prediction metrics [30]. The review not only discussed the applicability of fault prediction metrics, but also reported the datasets on which these metrics had been evaluated. It also tried to determine the correlation between the software development phase of the project and the fault prediction metric used. Similarly in this study, ECM and HCM are not only compared, but an effort is made to establish a relation between choice of entropy metric, that is, ECM (churn of entropy) and HCM (absolute entropy), and characteristics of the software system.

D’Ambros et al. conducted a study for evaluating various fault prediction approaches [6]. They not only evaluated the performance of different fault prediction metrics, but also provided a benchmark dataset for other researchers. It was concluded that the difference in results obtained by churn of source code and entropy metrics was not statistically significant. The main intuition for proposing churn of entropy is that degree of change in entropy will better model the faults rather than the absolute value of entropy. 

Canfora et al. empirically evaluated the relationship between complexity of source code and entropy of code changes based on four factors, namely, refactoring activities, number of developers working on a file, involvement of classes in design patterns, and type of change [31]. Their study tried to understand whether different types of changes produce a different magnitude of change in entropy. It was observed that for different types of changes, the difference in change in entropy was also statistically significant. In this study, ECM is used to model change in entropy, with an intuition that it is the change in entropy, rather than the absolute value of entropy, which leads to an indication of the number of faults in the software system.

Our study proposes a new fault prediction metric, that is, Entropy Churn Metric (ECM) based on HCM and churn of source code metric. It also compares the performance of the proposed metric (i.e., ECM with that of HCM). This will help determine whether it is better to use entropy of changes or churn of entropy of changes. The study not only proposes a new metric for fault prediction based on entropy of changes, but also investigates which metric is more suitable for what type of software systems. The software systems are characterized based on three parameters: (i) distribution of faults, (ii) system size, and (iii) programming language, to determine when it is preferable to use ECM over HCM and vice versa. 

## 3. Entropy Churn Metrics

Entropy Churn Metrics (ECM) are derived from History Complexity Metrics (HCM). ECM combines concepts of entropy of changes and churn of source code metrics. Before proposing the new metric for fault prediction (i.e., ECM), the following subsections describe HCM and churn of source code metrics.

### 3.1. History Complexity Metrics

HCM was given by Hassan [7]. Hassan measured the complexity of source code changes and quantified it using entropy of changes [7]. Entropy of changes for a particular time period (taken as one year for this study) is calculated using Shannon entropy [32], as defined in Equation (1).

(1) $${\mathrm{Entropy}}_{n}\left(P\right)=-\overset{n}{\underset{f=1}{\sum }}P_{f}\times \log_{2}P_{f}$$

where n denotes number of files in the software system and *P_f_* is the probability of changes in file *f* during the time period under consideration.

To account for different numbers of files in different software systems, the entropy defined in Equation (1) is normalized to give normalized entropy as defined in Equation (2).

(2) $$\mathrm{Normalized}\;\mathrm{Entropy}\left(P\right)=-\overset{n}{\underset{f=1}{\sum }}P_{f}\times \log_{n}P_{f}$$

This entropy of changes is used to calculate the History Complexity Metric (HCM). HCM of a file *b* is calculated using Equation (3).

(3) $${\mathrm{HCM}}_{\left\{i,\ldots ,j\right\}}\left(b\right)=\underset{k\epsilon \left\{i,\ldots ,j\right\}}{\sum }{\mathrm{HCPF}}_{k}\left(b\right)$$

where, {*i*,…,*j*} denotes the set of evolution periods and HCPF*_k_*(*b*) denotes the History Complexity Period Factor of file *b* for time period *k*. HCPF for file *b* is calculated using Equation (4).

(4) $${\mathrm{HCPF}}_{k}\left(b\right)=\{\begin{array}{cc} {\mathrm{Complexty}}_{kb}\times {\mathrm{Normalized}\;\mathrm{Entropy}}_{k} & ,b\;\epsilon \;M_{k}\\ 0 & \mathrm{otherwise} \end{array}$$

where Normalized Entropy*_k_* is the value of normalized entropy for the time period *k*, *M_k_* is the set of files modified in period *k*, and Complexity*_kb_* has the following three definitions:

- HCM1: Complexity*_kb_* = 1, Complexity associated with file *b* in period *k* is equal to one and HCPF is equal to the value of Normalized Entropy.
- HCM2: Complexity*_kb_* = *P_b_*, Complexity is equal to the probability of changes in file *b* in period *k* for files modified in that period. Otherwise, it is equal to zero.
- HCM3: Complexity*_kb_* = 1/|*M_k_*|, Complexity is equal to reciprocal of number of files modified in period *k* for files modified in that period. Otherwise, it is equal to zero.

HCM for a subsystem S over the set of evolution periods {*i*,…,*j*} is defined as the sum of HCM for all files in the subsystem as given in Equation (5).

(5) $${\mathrm{HCM}}_{\left\{i,\ldots ,j\right\}}\left(S\right)=\underset{b\epsilon S}{\sum }{\mathrm{HCM}}_{\left\{i,\ldots ,j\right\}}\left(b\right)$$

### 3.2. Churn of Source Code Metrics

Churn of source code metrics were first used by Nikora and Munson [18]. In [6], Dambros et al. compared this metric with other popular fault prediction metrics. The intuition behind use of churn of source code metrics was that it may provide better results than simple source code metrics like number of added and deleted lines of code. To calculate churn of source code metrics, the source code history needs to be sampled over every predefined period of time, say, two weeks, one month, or a year. If a class does notexist in a particular version, then its metric value is set to −1 (default value). The delta value of the source code metrics is calculated for each consecutive pair of samples using Equation (6).

(6) $$\mathrm{delta}\left(x,y\right)=\{\begin{array}{cc} -1 & \mathrm{if}\;v_{x}=0\;\mathrm{or}\;v_{y}=0\\ \left|v_{x}-v_{y}\right| & \mathrm{otherwise} \end{array}$$

where v*_x_* is the value of metric at sample *x* and v*_y_* is the value of metric at sample *y*; *x* and *y* are consecutive samples.

The churn value is then calculated using Equation (7).

(7) $$\mathrm{CHU}\left(x\right)=\overset{C}{\underset{y=1}{\sum }}\{\begin{array}{cc} 0 & \mathrm{delta}\left(x,y\right)=-1\\ \mathrm{delta}\left(x,y\right) & \mathrm{otherwise} \end{array}$$

where C is the number of samples. Thus churn is the summation of values of delta over all the samples excluding the samples where delta value is −1.

### 3.3. Entropy Churn Metrics (ECM)

The proposed Entropy Churn Metrics (ECM) enhance the History Complexity Metrics (HCM) by calculating the churn of entropy of code changes. This is done with the intuition that churn of entropy (i.e., churn of HCM) may provide better results than a simple entropy metric (i.e., HCM). The major motivation for proposing ECM is that, rather than absolute value of entropy, it may be the change in entropy that is a better predictor of faults. 

HCM and HCPF values for all files in the selected system/subsystem are computed using Equations (1)–(4). The Entropy Churn (ECHU) for a file *b* for period *j* is computed using Equation (8).

(8) $${\mathrm{ECHU}}_{j}\left(b\right)=\left|{\mathrm{HCM}}_{\left\{i,\ldots ,j+1\right\}}\left(b\right)-{\mathrm{HCM}}_{\left\{i,\ldots ,j\right\}}\left(b\right)\right|$$

where ${\mathrm{HCM}}_{\left\{i,\ldots j\right\}}\left(b\right)$ denotes the value of HCM metric of file *b* until period *j*. 

The ECM for a subsystem is calculated as the sum of ECHU for all files in the subsystem as depicted in Equation (9).

(9) $${\mathrm{ECM}}_{\left\{i,\ldots ,j\right\}}\left(S\right)=\underset{b\epsilon S}{\sum }{\mathrm{ECHU}}_{\left\{i,\ldots ,j\right\}}\left(b\right)$$

**Figure 1:**
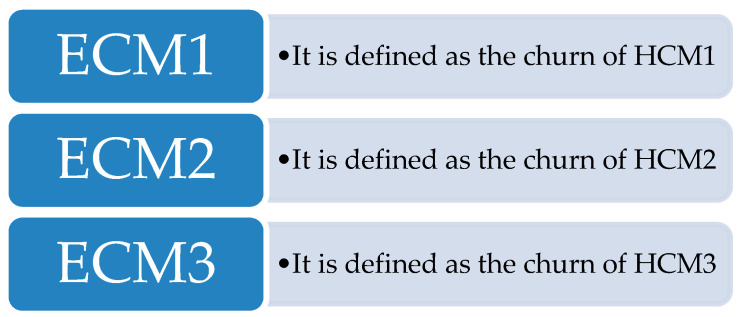
Three variants of ECM.

Similar to HCM, ECM has three variants, namely, ECM1, ECM2, and ECM3. The three variants of ECM are depicted in Figure 1.

In the next section, the detailed steps used for carrying out the research are described along with the datasets on which the ECM are evaluated.

## 4. Research Methodology

**Figure 2:**
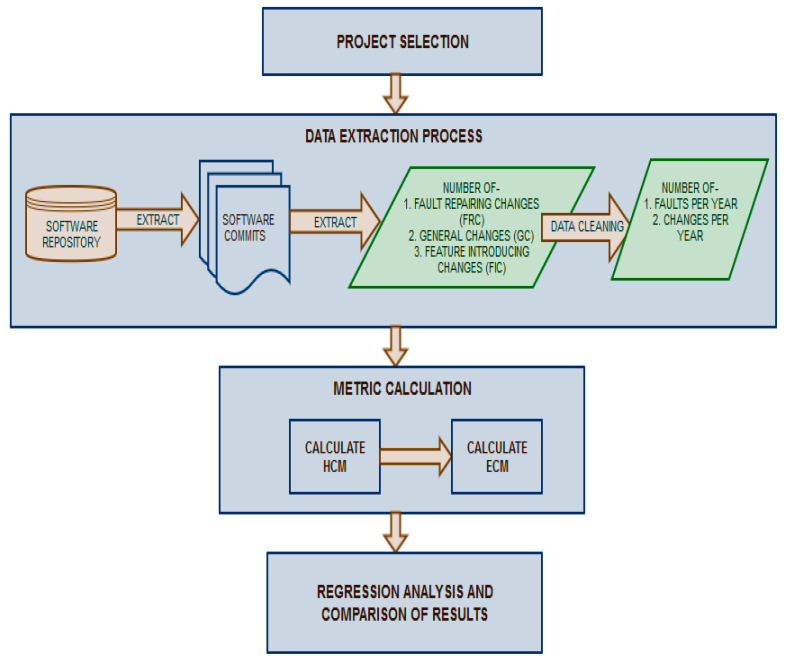
Research Methodology.

The process used for carrying out this study is depicted in Figure 2. The research was carried out in the following stages:

1. **Project Selection**: The first step was to determine the software projects and subsystems to be studied. The details of the software projects considered in this study are given in Section 4.1.
2. **Data Extraction**: The second step was to collect the number of changes per year and the number of faults per year in the selected subsystems of the software projects under study. This was done by first extracting the commits from software repositories and then analyzing the commits to determine the type of change done in the commit. The data regarding the number of changes of each type was then cleaned to determine the number of changes and faults per year. The data extraction process along with metric calculation is described in detail in Section 4.2.
3. **Metric Calculation**: The third step was metric calculation, which used the data collected in step two for calculating HCM and ECM. The data extraction and metric calculation processes are explained in Section 4.2.
4. **Regression Analysis and Comparison of Results:** In the next step, the two metrics (i.e., ECM and HCM) were compared for fault prediction using regression analysis. Finally, the results were analyzed and the study was concluded. The results are analyzed and discussed in Section 5 and Section 6, respectively.

### 4.1. Selected Software Projects and Subsystems

Five software projects were selected to conduct the study, namely, Android, Eclipse, Apache Http Server, Eclipse C/C++ Development Tooling (CDT), and Mozilla Firefox. Three subsystems each of Android, Apache Http Server, Eclipse C/C++ Development Tooling (CDT), and Mozilla Firefox were examined in the study and two subsystems of Eclipse were examined. The details of these subsystems and repositories are given in Table 1. Mozilla data was collected from Mozilla Central [33], whereas data for all other software projects was collected from GitHub [34]. These subsystems were chosen on the basis of following criteria:

- **Size and Lifetime:** The selected software subsystems included representatives from small-, medium-, and large-sized systems. The selected subsystems included both systems that had been released for several years as well as new systems that had been released only a few years ago. This criterion enabled us to determine the impact of size of the system on the prediction power of HCM and ECM.
- **Programming Language**: The selected software subsystems were programmed using different programming languages, namely, Java, C, and C++. Subsystems with different programming languages were selected so that the impact of programming language of the subsystem on prediction performance of ECM and HCM could be studied.
- **Availability of Data**: All data regarding the changes in the software subsystems was extracted from open source software repositories that are accessible to all.

### 4.2. Data Extraction and Metrics Calculation

Data required for computation of HCM and ECM was extracted from Mozilla Central and GitHub using a programmed tool [35]. The commits of each file in a subsystem were extracted using regular expression matching [36]. The extracted commits were then analyzed by the tool and the number of changes per year of each type was calculated. Similar to Hassan [7], the extracted changes are classified as follows:

- **Fault Repairing Changes (FRCs)**: the changes that are made to the software system for removing a bug/fault. These changes usually represent the fault resolution process and do not represent the code change process.
- **General Changes (GCs)**: the changes that are done for maintenance purposes and do not reflect any changed feature in the code. Examples of general changes include changes to copyright files and reindentation of the code.
- **Feature-Introducing Changes (FICs)**: the changes that are done with the intention of enhancing the features of the software system. These changes truly reflect the code change process.

The tool returned data regarding the number of changes per year of each type in a particular file of the software system. Hassan used the number of FRCs to validate the study, as they represent the fault resolution process rather than the code change process and hence do not impact the complexity of code changes [7]. The number of GCs was also not used to calculate the complexity of code changes as GCs are only maintenance-related changes that do not impact features of the code. The number of FICs were used to calculate the complexity of code changes, as these changes truly reflect the code change process. Similarly, in this study, only FICs were used to model the complexity of code changes, FRCs were used for validating the number of faults, and GCs were discarded. 

**Figure 3:**
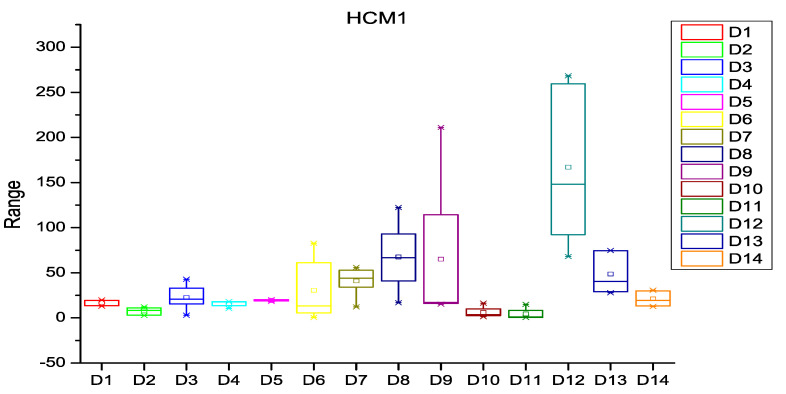
Box plot for HCM1.

**Figure 4:**
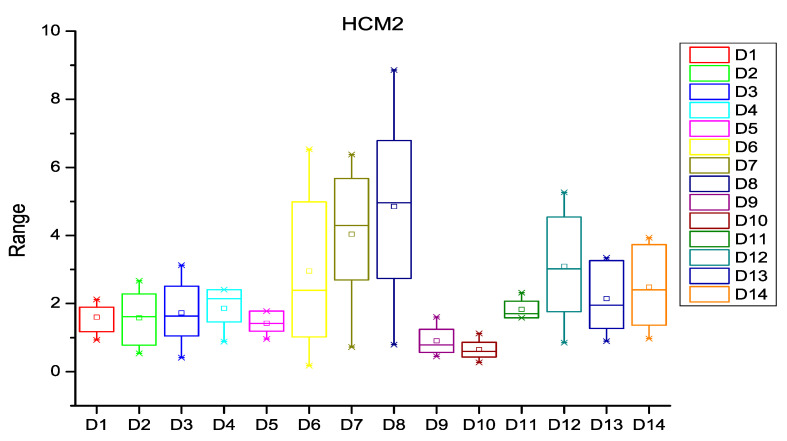
Box plot for HCM2.

**Figure 5:**
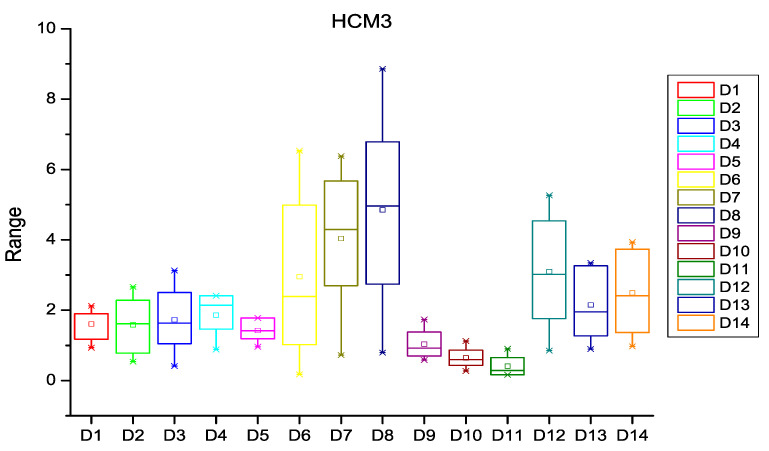
Box plot for HCM3.

**Figure 6:**
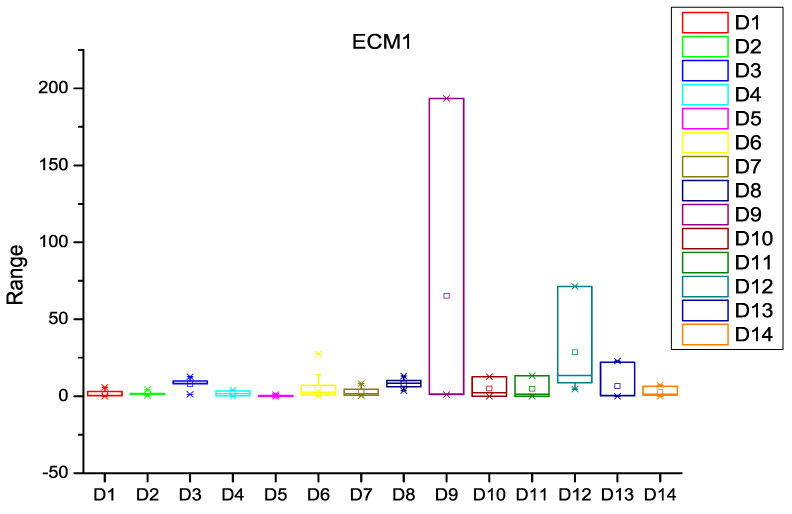
Box plot for ECM1.

**Figure 7:**
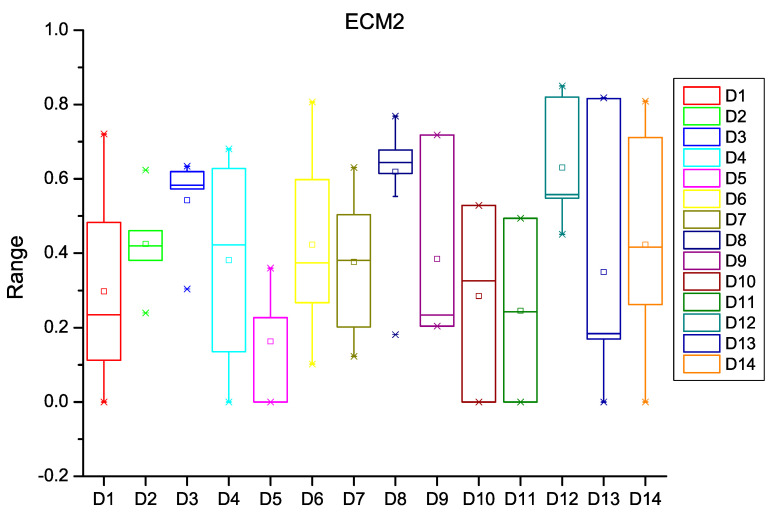
Box plot for ECM2.

**Figure 8:**
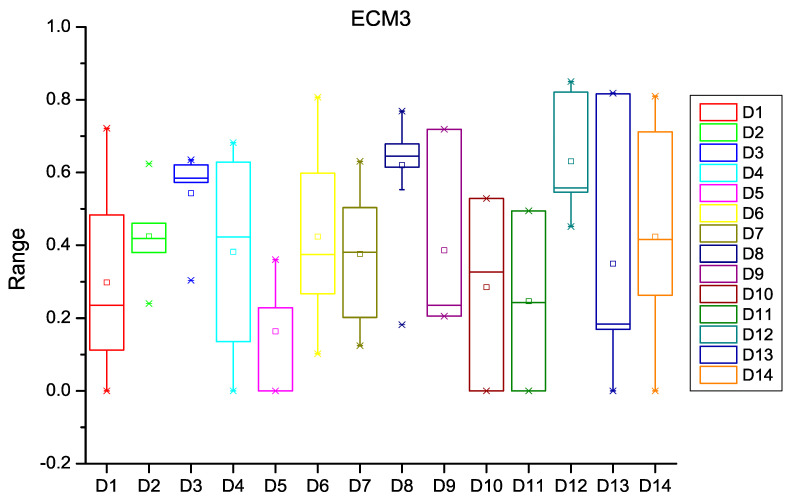
Box plot for ECM3.

After extracting the number of changes per year in each file of the software subsystem, the probability of change was calculated for every file of the subsystem. HCM and ECM were then calculated using Equations (1)–(5), (8) and (9) as described in Section 3. Figure 3, Figure 4 and Figure 5 depict the box plots for HCM1, HCM2, and HCM3 metrics, respectively, for the 14 selected datasets. ECM1, ECM2, and ECM3 metrics for the 14 datasets are depicted in Figure 6, Figure 7 and Figure 8.

## 5. Results

The performance of ECM was compared with that of HCM. Linear regressionwas employed for the comparison of prediction power of both these metrics [37]. Rapid Miner Studio [38] was used for performing linear regression with number of faults as the dependent variable and HCM/ECM metric as independent variable. The following parameters were used for the Linear Regression operation:

- Feature selection method: M5 Prime
- Eliminate collinear features: TRUE
- Minimum tolerance: 0.05
- Use bias: TRUE
- Ridge: 1.0 × 10^−8^

Table 2 lists the Root Mean Square Error (RMSE) [39] observed for each metric. 

The Friedman test was used to compare performance of the metrics over 14 datasets that were selected for this study [40]. The Friedman test ranks the performance of metrics for each dataset separately. The best performing metric gets rank one, the second best metric gets rank two, and so on; in case of ties, average rank is assigned. The results of the Friedman Test are depicted in Table 3. 

The Friedman test revealed that there was no statistically significant difference in prediction errors reported by the six metrics (Chi-Square= 10.190, significance = 0.070). Hence, it can be said that all the six metrics perform equally well for fault prediction, that is, there is no difference between ECM and HCM metrics. However, from Table 2, it can be observed that ECM performed better for 4 out of 14 datasets, HCM performed better for 6 out of 14 datasets, while both ECM and HCM gave equal results for 4 out of 14 datasets. This makes it essential to analyze the characteristics of the datasets in order to determine which metrics should be used with which datasets. For this purpose, we analyzed the datasets/software subsystems on the following parameters:

- Distribution of number of faults per year
- No. of files in the subsystem
- Programming language

These three parameters of the datasets and performance of ECM and HCM with respect to these parameters are analyzed in the following subsections.

### 5.1. Distribution of Faults

In this subsection, the datasets were classified based on whether the distribution of number of faults per year in a software subsystem was normal or non-normal. A dataset is said to be normally distributed if the curve is symmetric around the mean, otherwise, it has non-normal distribution. We used the Shapiro–Wilk Test to determine whether the distribution was normal or non-normal [41]. Table 4 depicts the distribution of number of faults for the 14 datasets.

It was observed that for normal distribution of number of faults per year, either of the two metrics may perform better. But when number of faults per year has a non-normal distribution, HCM always performs better than or equal to ECM. Hence, it can be concluded that HCM is better suited for subsystems having non-normal distribution of faults per year.

### 5.2. System Size

**Figure 9:**
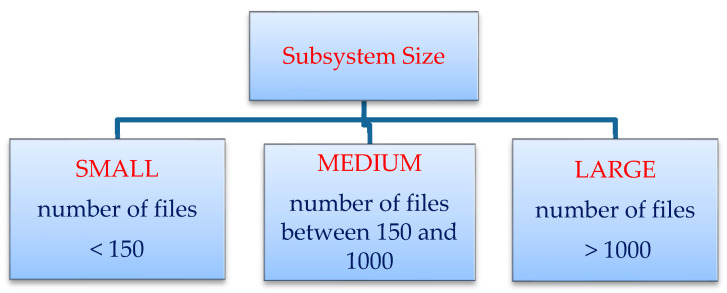
Classification of subsystems according to number of files.

A software subsystem/system can be classified according to the number of files in the subsystem. A subsystem can be classified as small, medium, or large according to the criteria shown in Figure 9. A subsystem is considered small-sized if the number of files in the subsystem is less than 150. It is considered to be medium-sized if the number of files in the subsystem is between 150 and 1000. A subsystem is considered large if it contains more than 1000 files. 

Table 5 specifies the classification of the 14 datasets based on size. It can be observed that for small-sized subsystems, HCM gave better or comparable results to ECM. But for medium-sized and large-sized subsystems, ECM provided better results. Hence, it can be inferred that for medium-sized and large-sized subsystems, it is better to use ECM, and for small-sized subsystems, it is better to use HCM. In other words, ECM is a better predictor when dealing with systems having large coupling between files.

### 5.3. Language

Programming language of the 14 datasets were analyzed in order to determine which metric (i.e., ECM or HCM) is better suited for software systems programmed using which language. The 14 datasets were programmed using C, C++, or Java, as shown in Table 6.

It can be observed that for subsystems programmed using C and C++, HCM performed better or comparable to ECM, whereas for software subsystems programmed using Java, either of ECM and HCM may give better results. Hence, it can be suggested that HCM should be preferred for software subsystems/systems programmed using C and C++.

## 6. Discussion

Comparison of ECM and HCM revealed that there is no statistically significant difference between the prediction errors obtained using both the metrics. This implies that both ECM and HCM have comparable performance and both of these metrics can be used for predicting faults in software systems.

In order to analyze which metric should be preferred for a particular software system, the results were analyzed based on three characteristics (distribution of faults, system size, and programming language) of the software system. The analysis of results leads to the following observations:

- It was observed that for normal distribution of faults, sometimes ECM performed better and sometimes HCM gave better results. But for non-normal distribution of faults, HCM always gave better or comparable results to ECM. Thus, while any of ECM and HCM may be used for prediction when using the distribution of faults, HCM should be the preferred metric when the distribution is non-normal.
- When the performance of HCM and ECM was analyzed with respect to the size of the system, it was observed that for small-sized systems, HCM gave better or comparable results to ECM, but for medium- and large-sized systems, ECM outperformed HCM. Thus it can be recommended to use ECM for medium- and large-sized systems and HCM for small-sized systems.
- Lastly, on analyzing the performance of ECM and HCM with regard to the programming language of the system, it was observed that when the programming language of the subsystem was C/C++, the results obtained using HCM were always better or comparable to those obtained using ECM. It was also observed that when the programming language of the system was Java, either of ECM and HCM gave better results. Hence, it can be suggested that for systems programmed in C/C++, the preferred metric should be HCM, while for systems programmed in Java, either of ECM and HCM may be preferred.

## 7. Threats to Validity

Fault prediction studies are predisposed to factors that influence the accuracy and reliability of the results. These are called threats to validity of a study. Comprehensively, there are two types of threats to validity: internal and external threats. 

### 7.1. Threats to Internal Validity

Threats to internal validity happen if there is error in representation of causes that influence the outcomes of the study. One such validity threat is that this study uses FRCs for validating the study. The study does not contemplate the faults that are reported but not removed while counting the year-wise number of faults. Since there are scarcely any faults that are not removed, the results of this study are justified. 

Another concern is regarding accuracy of classification of changes. The tool used for this purpose is not artificially intelligent, that is, it does not exhibit human-like intelligence and relies on simple rules to determine the reason of change for the commits extracted from the software repository. However, the tool utilizes a basic yet powerful keyword-matching algorithm to classify the commits with minimum odds of misclassification. 

### 7.2. Threats to External Validity

Threats to external validity refer to concerns associated with generalization of the findings of the study. For this reason, even though the study was conducted on 14 software subsystems from five different software projects, the study should be replicated for other software systems also. 

Another threat to validity is that although the study was conducted on 14 subsystems of different size, of different programming language, and having different distribution of faults, they are from open source software projects only, and these subsystems cannot be considered as representations of subsystems of industrial software projects. Further studies should be carried out to check the applicability of ECM for industrial software projects also.

## 8. Conclusion

The paper proposed a new metric for fault prediction based on churn of entropy (i.e., ECM). ECM was compared with HCM for 14 software subsystems. The subsystems were selected from popular software projects: Android, Eclipse, Apache Http Server, Eclipse C/C++ Development Tooling (CDT), and Mozilla Firefox. The results revealed that there was no statistically significant difference between the performance of HCM and ECM. Thus both the metrics can be used for fault prediction in software systems by analyzing its change history. 

While ECM gave better results for 4 out of 14 datasets, HCM gave better results for 6 out of 14 datasets, and both ECM and HCM gave equal results for 4 out of 14 datasets. The study further analyzed the characteristics of the software subsystems in order to determine which metrics were better suited for what type of software systems. The subsystems were analyzed based on three parameters, namely, (a) distribution of number of faults per year, (b) number of files, and (c) programming language. It was inferred from the analysis that, (a) HCM metric are more appropriate for software systems/subsystems having a non-normal distribution of faults per year, (b) ECM metrics are better suited for medium-sized and large-sized software systems/subsystems, and (c) HCM metrics are a better choice for software subsystems/systems programmed using C and C++.

A major conclusion that can be drawn from the study is that entropy metrics are dependent on subsystem size. Simple entropy metrics (i.e., HCM) are better predictors of faults than ECM when the system size is small. As the system sizes increases, the coupling between files becomes large and it becomes difficult for programmers to efficiently organize the files. It was observed that under such circumstances (i.e., large-sized systems with large coupling), ECM or churn of entropy is a better predictor of faults. However, further studies specially using proprietary software projects and a comparison with popular fault prediction metrics other than HCM should be done to explore the applicability of ECM.

## Figures and Tables

**Table 1:** Software Projects and Subsystems studied.

Software Project	Repository	Subsystem	No. of Files	URL	Acronym
Android	GitHub	android/platform frameworks base/drm	17	https://github.com/android/platform_frameworks_base/tree/master/drm	D1
		android/platform frameworks base/keystore	18	https://github.com/android/platform_frameworks_base/tree/master/keystore	D2
		android/platform frameworks base/location	80	https://github.com/android/platform_frameworks_base/tree/master/location	D3
Eclipse	GitHub	eclipse/platform-core	196	https://github.com/eclipse/eclipse/tree/master/platform-core	D4
		eclipse/development	282	https://github.com/eclipse/eclipse/tree/master/development	D5
Apache Http Server	GitHub	apache/httpd/modules/filters	61	https://github.com/apache/httpd/tree/trunk/modules/filters	D6
		apache/httpd/modules/mappers	38	https://github.com/apache/httpd/tree/trunk/modules/mappers	D7
		apache/httpd/modules/ ssl	34	https://github.com/apache/httpd/tree/trunk/modules/ssl	D8
Eclipse C/C++ Development Tooling (CDT)	GitHub	eclipse/cdt/build	2063	https://github.com/eclipse/cdt/tree/master/build	D9
		eclipse/cdt/codan	385	https://github.com/eclipse/cdt/tree/master/codan	D10
		eclipse/cdt/dsf	745	https://github.com/eclipse/cdt/tree/master/dsf	D11
Mozilla Firefox	Mozilla Central (Mercurial Repository)	mozilla-central/layout/ generic	132	http://hg.mozilla.org/mozilla-central/file/tip/layout/generic	D12
		mozilla-central/layout/ forms	43	http://hg.mozilla.org/mozilla-central/file/tip/layout/forms	D13
		mozilla-central/layout/ printing	14	http://hg.mozilla.org/mozilla-central/file/tip/layout/printing	D14

**Table 2:** RMSE of each metric.

Dataset	HCM1	HCM2	HCM3	ECM1	ECM2	ECM3
D1	2.757	**1.396**	**1.396**	11.500	11.500	11.500
D2	4.680	**4.261**	**4.261**	7.658	7.658	7.658
D3	**17.691**	**17.691**	**17.691**	**17.691**	**17.691**	**17.691**
D4	**1.920**	**1.920**	**1.920**	**1.920**	**1.920**	**1.920**
D5	2.059	2.059	2.059	**1.359**	2.059	2.059
D6	**15.756**	**15.756**	**15.756**	**15.756**	**15.756**	**15.756**
D7	**13.877**	16.990	16.990	23.356	25.748	25.748
D8	42.682	**42.270**	**42.270**	46.546	46.546	46.546
D9	381.786	381.786	381.786	76.917	**57.880**	**57.880**
D10	69.801	69.801	69.801	**5.711**	37.700	37.700
D11	12.656	12.656	12.656	**3.856**	41.272	41.272
D12	140.827	**137.312**	**137.207**	451.133	451.133	451.133
D13	74.449	**66.326**	**66.326**	161.429	161.429	161.429
D14	**30.223**	**30.223**	**30.223**	**30.223**	**30.223**	**30.223**

**Table 3:** Results of Friedman Test.

Test Statistics	
N	14
Chi-Square	10.190
df	5
Asymptotic Significance	0.070

**Table 4:** Distribution of faults per year.

Dataset	Distribution
D1	Normal
D2	Normal
D3	Normal
D4	Normal
D5	Normal
D6	Normal
**D7**	**Non-normal**
**D8**	**Non-normal**
D9	Normal
D10	Normal
D11	Normal
D12	Normal
D13	Normal
**D14**	**Non-normal**

**Table 5:** Classification of subsystems based on size.

Dataset	No. of Files	Subsystem Size
D1	17	Small
D2	18	Small
D3	80	Small
**D4**	**13**	**Medium**
**D5**	**21**	**Medium**
D6	61	Small
D7	38	Small
D8	34	Small
**D9**	**2063**	**Large**
**D10**	**385**	**Medium**
**D11**	**745**	**Medium**
D12	132	Small
D13	43	Small
D14	14	Small

**Table 6:** Programming language of the software subsystems.

Dataset	Programming Language
D1	JAVA
D2	JAVA
D3	JAVA
D4	JAVA
D5	JAVA
**D6**	**C**
**D7**	**C**
**D8**	**C**
D9	JAVA
D10	JAVA
D11	JAVA
**D12**	**C++**
**D13**	**C++**
**D14**	**C++**
